# Vitamin B12 in Cats: Nutrition, Metabolism, and Disease

**DOI:** 10.3390/ani13091474

**Published:** 2023-04-26

**Authors:** Gerardo Siani, Beatrice Mercaldo, Maria Chiara Alterisio, Antonio Di Loria

**Affiliations:** 1 DVM, 84133 Salerno, Italy; info@drsiani.com; 2 Department of Veterinary Medicine and Animal Productions, University Federico II of Napoli, Via F. Delpino 1, 80137 Napoli, Italy; beatrice.mercaldo@unina.it (B.M.); mariachiara.alterisio@unina.it (M.C.A.)

**Keywords:** cobalamin, cat, methylmalonic acid

## Abstract

**Simple Summary:**

We review the role of vitamin B12 in cats. Cobalamin is an essential part of enzymes involved in multiple metabolic reactions. In mammals, two enzymes are cobalamin-dependent: methionine synthase and methylmalonyl-CoA mutase. Cats are obligate carnivores and have an essential requirement for certain nutrients present only in animal tissues, including vitamin B12, which cannot be synthesized by cats. The serum cobalamin concentration can be measured by an automated chemiluminescence competitive binding immunoassay system but does not accurately reflect the real availability of cobalamin, because cobalamin-dependent metabolic reactions occur within cells. A lack of intracellular cobalamin leads to reduced enzyme activity and methylmalonic acid accumulation. The serum cobalamin concentration is particularly useful for detecting deficiency, as it is directly related to the real bioavailability of the vitamin for intracellular enzymatic reactions. Several diseases of the gastrointestinal tract, pancreas, and liver are associated with hypocobalaminemia in cats. Treatment with parenteral cobalamin is always recommended for cats with gastrointestinal disease and low serum cobalamin concentrations. A recognized guideline for cobalamin supplementation does not yet exist for cats, so the recommendations are mainly based on clinical experience.

**Abstract:**

Cobalamin is a water-soluble molecule that has an important role in cellular metabolism, especially in DNA synthesis, methylation, and mitochondrial metabolism. Cobalamin is bound by intrinsic factor (IF) and absorbed in the ileal tract. The IF in cats is synthesized exclusively by pancreatic tissue. About 75% of the total plasma cobalamin in cats is associated with transcobalamin II, while in this species, transcobalamin I is not present. In cats, the half-life of cobalamin is 11–14 days. Diagnostic biomarkers for B12 status in cats include decreased levels of circulating total cobalamin and increased levels of methylmalonic acid. The reference interval for serum cobalamin concentrations in cats is 290–1500 ng/L, and for the serum methylmalonic acid concentration, it is 139–897 nmol/L. Therapy for hypocobalaminemia mainly depends on the underlying disease. In some cases, subcutaneous or intramuscular injection of 250 μg/cat is empirically administered. In recent years, it has been demonstrated that oral cobalamin supplementation can also be used successfully in dogs and cats as a less invasive alternative to parental administration. This review describes the current knowledge regarding B12 requirements and highlights improvements in diagnostic methods as well as the role of hypocobalaminemia in its associated diseases.

## 1. Introduction

**Figure 1:**
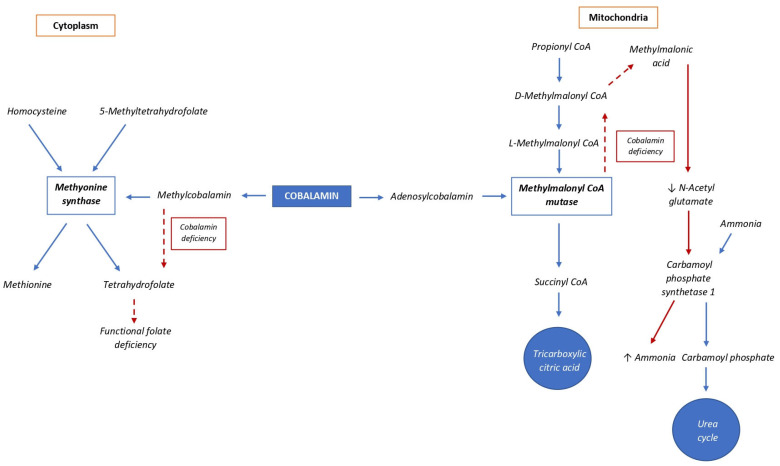
Cytoplasmic and mitochondrial enzymatic reactions of cobalamin. Blue arrows indicate reactions that normally occur in presence of cobalamin; dashed red arrows indicate changes due to cobalamin deficiency; solid red arrows indicate the consequences of methylmalonic acid increase.

Cobalamin is a water-soluble, cobalt-containing B vitamin [1] resulting from microbial synthesis, mainly bacteria, present in the rumen and intestine, as well as soil [2]. This vitamin is an essential catalyzer for nucleic acid synthesis and hematopoiesis [3]. In mammalian species, only two enzymes are known to be cobalamin-dependent: (i) methionine synthase, a methyl transferase, and (ii) methylmalonyl-CoA mutase, an isomerase. Methionine synthase catalyzes the transfer of a methyl group to S-adenosyl-homocysteine, generating S-adenosyl-methionine and hence methionine, while methylmalonyl-CoA mutase catalyzes the isomerization of methylmalonyl-CoA to succinyl-CoA, which then enters the citric acid cycle (Figure 1) [4,5,6]. The major food sources of cobalamin are animal products; plant products essentially lack this vitamin [2,7]. Cobalamin is the only B vitamin not present in plant materials [7]. In veterinary medicine, information on cobalamin metabolism, requirements, and therapy in cats appears to be poor and incomplete. The purpose of this study was to summarize the current knowledge on vitamin B12 in cats, including dietary recommendations, metabolism, and assessment of cobalamin status and its deficiency, especially related to diseases.

## 2. Cobalamin in Cats’ Diet

Cats are obligate carnivores; therefore, they have an essential requirement for nutrients present only in animal tissues [8]. Vitamin B12 is among these essential nutrients because it cannot be synthesized by cats. However, it would appear that a small amount of cobalamin can be synthesized by the intestinal flora present in an area unable to absorb it [2], therefore making the vitamin unavailable. Despite the crucial role of cobalamin, there are currently no studies assessing minimum requirements for weaned kittens, adult cats, or breeding and lactating queens. An amount of 4.5 µg per 1000 Kcal of metabolizable energy (ME), administered through purified diets, was able to maintain normal hemoglobin concentrations in growing kittens [9]. When minimum requirements have not been defined for dietary nutrients, adequate intake can be presumed as the concentration in the diet required to sustain a given life stage [2]. An adequate intake of 4.5 µg per 1000 Kcal ME is suggested, although the NRC recommends including a safety factor of 5.6 µg per 1000 Kcal ME [2]. The European Pet Food Industry Federation (FEDIAF) recommends a dietary cobalamin intake of 4.5 µg per 1000 Kcal ME for breeding kittens and 4.4 µg per 1000 Kcal ME for adult cats [10]. For cat growth, breeding, and adult maintenance, the American Association of Feed Control Officials (AAFCO) recommends a dietary cobalamin intake of 4.5 µg per 1000 Kcal ME [11].

Neither a safe upper limit of cobalamin (the maximum concentration in the diet or an amount that has been associated with adverse effects) or reports of toxicity as a result of cats ingesting high doses are present in the literature [2]. Dietary intake of vitamin B12 is essentially provided by products of animal origin (i.e., meats), which largely represent the principal part of this species’ obligate carnivore diet [8]. Thus, it is unlikely that deficiencies would develop in healthy and well-fed cats. Cobalamin is considered very stable in foodstuffs [2]; however, it is thermolabile. Cooking can inactivate it, depending on the temperature and the type of food [12,13,14]. Homemade cooked diets should be supplemented with vitamin B12 accordingly, while raw diets should not be provided unless the recommended intake is achieved. While vitamin B12 content in food for human consumption is well reported [15], it is not well described in pet food. As in other developed economic areas, European pet food companies are not required to declare the analytical or additive value of vitamin B12 on product labels [16]. The dietary source of cobalamin in pet foods has historically been animal products, although most pet food contains bioavailable synthetic cobalamin produced by microbial fermentation. For plant-based diets, appreciated by vegan and vegetarian pet owners, the addition of synthetic cobalamin can meet the animals’ dietary requirement for the vitamin [7].

## 3. Metabolism

**Figure 2:**
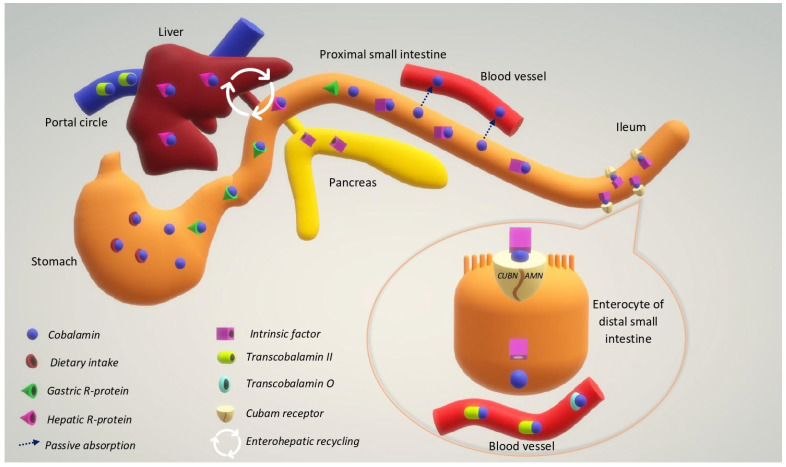
Mechanism of cobalamin absorption, transport, and enterohepatic recirculation. CUBN, cubilin subunit of cubam receptor; AMN, amnionless subunit of cubam receptor.

After food is ingested, cobalamin is released in the stomach by peptic digestion and bound to a gastric R-protein (also called haptocorrin) to form an unattackable complex [2]. Gastric R-protein protects cobalamin from bacteria within the proximal gastrointestinal tract. Pancreatic enzymes (trypsin and chymotrypsin) digest the gastric R-protein in the small intestine, and the free cobalamin (extrinsic factor) is bound by intrinsic factor (IF) to be absorbed in the ileal tract (Figure 2). The IF appears to be species-specific [17]: in dogs it is synthesized by pancreatic tissue and to a lesser extent by the gastric mucosa [17,18,19], while in cats it is synthesized exclusively by pancreatic tissue [18,19,20,21]. The cobalamin–IF complex is a stable compound that can bind to specific receptors located on the microvilli of ileal enterocytes [22]. The ileum is generally regarded as the major site of cobalamin absorption in mammals [2], but significant absorption was also shown to occur in the jejunum of cats and dogs [23].

The IF–cobalamin complex enters the enterocytes by pinocytosis after binding to specific receptors known as cubam receptors. They are receptor complexes consisting of two subunits, cubilin (CUBN) and the protein amnionless (AMN) [24,25]. Cobalamin is separated from IF by lysosomal enzymes and released into the circulation for transport to target tissues [26]. The plasma and extracellular fluid include transport proteins that bind cobalamin: about 75% of total plasma cobalamin in dogs and cats is associated with transcobalamin II. Cat plasma also contains transcobalamin O, while it lacks transcobalamin I, which is present in dog and human plasma [27]. Transcobalamin O carries about 10 to 15% of the circulating cobalamin [28]. Enterohepatic recycling has also been described in cats, in which cobalamin binds to a hepatic R-protein and is then secreted through the bile [29]. In this species, cobalamin appears mainly in the form of adenosylcobalamin and hydroxocobalamin [26]. In humans, the half-life of cobalamin is approximately one year, and in the case of cobalamin malabsorption, clinical signs occur after several years. In dogs, the half-life is around 42–112 days, while in cats it is 11–14 days [30]. Moreover, cats have a lower capacity to store cobalamin than humans and they became hypocobalaminemic within a month [31]. Although placental transfer of cobalamin has been reported in dogs [32], there are no studies describing this in cats.

Free cobalamin is stored in the liver and kidney [17] and is excreted through the kidney. The transcobalamin II–cobalamin complex undergoes renal glomerular filtration and subsequent tubular reabsorption. Megalin and cubam receptors are located at the renal tubule and show high affinity for the transcobalamin II–cobalamin complex [33,34].

## 4. Evaluation of Cobalamin Status and Deficiency in Cats

The serum cobalamin concentration is measured by an automated chemiluminescence competitive binding immunoassay system [35,36,37]. The reference interval for feline serum cobalamin concentration is 290–1500 ng/L and values <290 ng/L are considered subnormal [35,37]. A serum cobalamin concentration <100 ng/L represents the lower limit of detection of the chemiluminescent assay system for cats [29,37]. For serum cobalamin assessment, samples should be promptly stored at −20 °C. However, this vitamin remains stable for 5 days if samples are refrigerated at 6 °C even if they are not protected from light [17,38].

The direct influence of diet on serum cobalamin concentration can depend on several factors, and age, sex, and breed play important roles. Older cats frequently show decreased absorption of this vitamin, probably related to a decreased ability to digest it due to reduced ileal and pancreatic function [39,40]. Kittens show lower serum cobalamin concentration during the first months of life; this may be related to the immaturity of the microbiota, the gastrointestinal tract, and the pancreas. A gradual increase in cobalamin serum level is observed in young cats during the first year of age, when microbiota, gastrointestinal tract, and pancreas complete their development [41]. Male cats usually show significantly higher serum cobalamin concentrations than females [39]. Higher concentrations of vitamin B12 have been recorded in pure-breed cats than mixed-breed cats [42,43]. Barron et al. (2009) reported that the median serum cobalamin concentration in healthy cats is lower in older and mixed cats, but not in male cats, contrary to what was suggested by Hill et al. (2018) [44].

The serum cobalamin concentration does not accurately reflect the real availability of cobalamin, because cobalamin-dependent metabolic reactions occur within cells, mainly in the cytoplasm and mitochondria [15]. Methylmalonyl-CoA mutase catalyzes the formation of succinyl-CoA from methylmalonyl-CoA, which is produced by the catabolism of odd-chain fatty acids and amino acids. Succinyl-CoA is a key molecule in the citric acid cycle. A lack of intracellular cobalamin leads to reduced enzyme activity and an intracellular and subsequent systemic accumulation of methylmalonic acid (MMA), resulting in methylmalonic acidemia [29]. This molecule can also inhibit the activity of carbamoyl phosphate synthetase I, an enzyme of the urea cycle that normally metabolizes ammonia to carbamoyl phosphate. When this metabolic process is impaired, plasma ammonia concentrations typically increase [17] and neurological disorders can occur [45]. Therefore, the MMA concentration in cat serum is particularly useful for detecting cobalamin deficiency, as it is directly related to the real bioavailability of the vitamin for intracellular enzymatic reactions [46].

Serum and urinary MMA concentrations can be determined by gas chromatography–mass spectrometry in fresh or stored samples (−20 °C) [17,35,37]. The reference interval of serum MMA concentration defined for cats is 139–897 nmol/L [29]. The limitation of MMA measurement is mainly the higher cost compared to cobalamin detection [15]. Cats with severely subnormal serum cobalamin concentrations typically show extremely elevated serum MMA concentrations; a 50-fold increase over the highest normal value has been documented [46]. Although the serum MMA concentration reflects an intracellular cobalamin deficiency, other causes of elevated MMA must be considered, such as renal failure, reduced plasma volume reduction, and abnormalities in hepatic methyl-malonyl CoA mutase activity [15,47]. In both dogs and cats, normal serum MMA concentration with low serum cobalamin status seems to be a possible clinical finding. Low serum cobalamin levels could reflect depletion and redistribution of body reserves prior to real cobalamin deficiency in the mitochondria [37,48,49]. There is a lack of available data regarding urinary MMA determination in cats. To reduce the dilution effect and the influence of renal impairment, the urinary MMA-to-creatinine ratio should be considered. In cats, the reference range has been defined as 0.22–0.51 mmol/mol creatinine [3]. While a correlation between serum cobalamin deficiency and increased urinary MMA concentration has been observed in cats, no significant correlation has been detected between serum and urinary MMA concentrations [3,50]. In dogs, measuring urinary MMA concentration appears to be more advantageous due to its high stability in urine; in this species, urinary levels appear to be more concentrated than serum levels (urinary MMA concentration can be up to 40 times higher than serum concentration) [17].

In humans, total plasma homocysteine levels are used in the diagnosis of cobalamin deficiency. Cobalamin represents an essential cofactor for the enzyme methionine synthase, which is essential for the synthesis of methionine from homocysteine. Cobalamin deficiency blocks this reaction by promoting homocysteine accumulation. When not enough cobalamin is available, the pathway is blocked and homocysteine accumulates within the cells and plasma [51]. In humans, hyperhomocysteine is related to cobalamin deficiency [52], whereas in dogs, homocysteine can be used mainly in the diagnosis of familiar cobalamin deficiency specifically in the Chinese Shar-Pei [53], but only after other diseases have been ruled out [54]. In cats, homocysteine is not used as a diagnostic marker [15,55], because cobalamin deficiency is not associated with hyperhomocysteinemia in this species [29,30,37]. Even when the serum cobalamin concentration is extremely low or undetectable, the homocysteine level will fail to diagnose cobalamin deficiency probably because methionine is commonly added to commercial diets for cats, since it is generally the most limited amino acid in their diet [29]. A recent study evaluated serum cobalamin concentrations in healthy tigers (*Panthera tigris*) and tigers affected by pancreatic insufficiency-like syndrome. The results showed much lower B12 concentrations in both healthy and affected tigers than in domestic cats, although reference ranges for serum cobalamin concentrations in tigers have not yet been established [56]. Another study was conducted on cheetahs (*Acinonyx jubatus*) to establish reference ranges of species-specific biomarkers of gastrointestinal disease, including cobalamin and MMA. The measured ranges were 470–618 ng/L and 365–450 nmol/L for cobalamin and MMA, respectively, showing that even between closely related species such as the domestic cat and cheetah, there can be differences in gastrointestinal biomarkers [57].

### 4.1. Interpretation of Cobalamin Status and Diseases

Cobalamin status can be affected by various diseases either directly (e.g., by affecting its absorption and/or metabolism) or indirectly (e.g., by interfering with its intake). A typical example of the latter is anorexia, a condition that frequently occurs in disease states associated with hypocobalaminemia. Indeed, in cats, a low capacity for storage and the short half-life of cobalamin predispose them to develop B12 deficiency in a short time. Therefore, anorexia should always be considered as a cause of hypocobalaminemia in sick cats. The pathological conditions related to hypocobalaminemia that have a direct influence are described below.

#### 4.1.1. Hypocobalaminemia

Several diseases of the gastrointestinal tract, pancreas, and liver are associated with hypocobalaminemia in cats. In this species, inflammatory bowel disease (IBD), alimentary lymphoma, pancreatitis, cholangitis/cholangiohepatitis [29,30,35], and exocrine pancreatic insufficiency (EPI) [55] are the diseases frequently associated with cobalamin deficiency [29,30,35]. In particular, EPI can lead to a lack of IF and consequent hypocobalaminaemia [35], whereas alimentary lymphoma and chronic intestinal inflammation are responsible for impaired nutrient uptake [30,37].

Rapidly dividing cells such as enterocytes and hematopoietic cells, which are essential for DNA replication, are more susceptible to decreased serum cobalamin concentrations [39]. Other diseases characterized by increased metabolism (e.g., hyperthyroidism) are associated with decreased serum cobalamin concentrations [58]. Older cats with intestinal, pancreatic, or hepatobiliary disease or in an anorexic state are at greater risk of cobalamin deficiency, because serum cobalamin concentration physiologically decreases with age [39]. While congenital breed-related dysfunctions of the cubam receptors are proven in dogs [59,60], a similar predisposition is not described in cats [35,46].

Clinical signs closely related to cobalamin deficiency in cats have not been well characterized because hypocobalaminemia is often associated with other pathologies [46]; frequently described clinical signs include anorexia, vomiting, diarrhea, growth disorders, and neurological findings. Under cobalamin deficiency, non-regenerative anemia, leukopenia, hypoglycemia, and hyperammonemia seem to be the most frequent hematobiochemical alterations [15,61]. Hypocobalaminemia can interfere with hematopoiesis and can be responsible for anemia and pancytopenia [46,61].

#### 4.1.2. Hypercobalaminemia

Increased serum cobalamin may be indicative of liver disease or neoplastic disease [43,62]. The most common neoplasias associated with hypercobalaminemia in cats are lymphoma, pancreatic carcinoma, biliary cystadenoma, and metastatic splenic tumors [43], but the reasons for the correlation between hypercobalaminemia and neoplastic conditions in cats are still unknown. Sysel et al. (2015) found that the expression of transcobalamin II and its cell surface receptors was significantly higher in various tumor tissues than in corresponding adjacent normal tissues in both dogs and cats [63]. Although less frequent, hypercobalaminemia in cats has been also recorded during chronic enteropathy, acute or chronic pancreatitis, cholangiohepatitis, gastric lymphoma, and hyperthyroidism [62]. Hypercobalaminemia can be found in cats that recently received supplementation [43].

#### 4.1.3. Gastrointestinal Disease

Since cobalamin–IF complex receptors are located in the ileum, chronic gastrointestinal disease, alimentary lymphoma, and ileum resection can lead to cobalamin deficiency, as these conditions reduce ileum absorption capacity [35]. Hypocobalaminemia in gastrointestinal diseases could also be due to concurrent conditions such as pancreatic or hepatobiliary disease, or a combination of these [30], and several studies confirm the correlation between intestinal, liver, and pancreatic diseases and hypocobalaminemia [64,65,66]. Kiselow et al. (2008) and Kook et al. (2012) reported that gastrointestinal lymphoma could lead to markedly subnormal serum cobalamin concentrations (<150 ng/L) [65,67]. Gianella et al. (2017) reported that serum cobalamin levels were lower in cats with alimentary lymphoma than those with food-responsive enteropathy or idiopathic inflammatory bowel disease [68]. Different rates of hypocobalaminemia in cats affected by lymphoma of the small intestine have been reported: in Kieselow et al.’s study it was 78%; in Kook et al.’s study it was about 69%; and Jugan and August (2017) reported a rate of 42%. The prevalence of hypocobalaminemia in cats affected by IBD was found to be between 43% [65] and 47% (Jugan & August, 2017). Cats with gastrointestinal lymphoma and normal serum cobalamin survive longer than those with hypocobalaminemia [67]. Especially, the serum concentration of cobalamin decreases significantly [69] when there is a diagnosis of IBD or dietary lymphoma compared with gastrointestinal neoplasia. A correlation has been shown between clinical signs of gastrointestinal disease and intestinal pathology and reduced cobalamin [30,65]. In most healthy cats, cobalamin concentrations are ≥500 ng/L [29,35,37], but in most cats with gastrointestinal disease the concentrations are <500 ng/L [30]. Consequently, in all cats with diagnosed or suspected gastrointestinal disease, cobalamin status should be assessed. In addition, gastrointestinal malabsorption can be responsible for both cobalamin and iron deficiency, resulting in anemia. An evaluation of iron status and MMA should be included in the diagnostic pathway for cats with chronic gastrointestinal disease [70].

#### 4.1.4. Exocrine Pancreatic Insufficiency

Several studies have reported that 100% of cats with EPI show hypocobalaminemia [50,71,72], because the exocrine pancreas is the only site of IF synthesis in cats. EPI is diagnosed by measuring the concentration of feline serum trypsin-like immunoreactivity (fTLI); a value less than 8 μg/L indicates exocrine pancreatic insufficiency [73]. Cats with cobalamin deficiency show significantly lower serum fTLI concentrations than cats with normal cobalaminemia [74]. This suggests that normocobalaminemic cats with EPI have milder or early-stage disease, because fTLI indicates the functional capacity of the exocrine pancreas. The clinical condition is improved after appropriate treatment for EPI and cobalamin supplementation [73,74] in both hypocobalaminemic and normocobalaminemic cats [74]. Cats that do not respond appropriately to enzyme and cobalamin supplementation may have concurrent small intestinal disease, such as IBD [71].

#### 4.1.5. Pancreatitis and Triaditis

Triaditis in cats is described as concurrent inflammation of the pancreas, liver, and small intestine caused by bacterial infection or by immune-mediated or idiopathic mechanisms [75]. Pancreatitis in cats is often associated with other diseases (IBD, hepatic lipidosis, diabetes mellitus, intestinal lymphoma, and nephritis) and causes vitamin deficiencies; in these cases, cobalamin supplementation may be necessary [30].

#### 4.1.6. Dysbiosis

Dysbiosis resulting from EPI, gastritis, or other gastrointestinal disorders [35] may contribute to cobalamin deficiency. The abnormal presence of cobalamin-binding bacteria (e.g., *Bacteroides* spp., *Clostridium* spp.) may reduce its bioavailability [15]. The metabolism of vitamin B12 provides a protective system, such as gastric and hepatic R-proteins and IF, to avoid the utilization of bacteria. However, some bacteria are able to bypass the protective binding IF, as in the case of Gram-negative anaerobes of the genus Bacteroides [76]. In humans, cobalamin availability has been shown to influence the alpha and beta diversity of bacteria, the amount of bacteria, and their function in the gut [77]. In addition, the gut microbiome possesses genes coding for cobalamin transporters [78].

#### 4.1.7. Hepatic Lipidosis

Feline hepatic lipidosis is a common syndrome in cats due to fatty degeneration of hepatocytes with resulting cholestasis and hepatic dysfunction [64]. This syndrome can be idiopathic or secondary to pathological conditions that induce anorexia and subsequent alteration in triglyceride metabolism. Hepatic lipidosis is associated with several deficiencies, including glutathione, taurine, vitamin K1, and cobalamin [64]. An evaluation of serum cobalamin concentrations in cats with lipidosis revealed that 40% had subnormal levels [30]. Cobalamin deficiency contributes to anorexia and can predispose cats with other diseases to develop hepatic lipidosis. Beyond anorexia, the compression exerted by triglyceride-infilled hepatocytes on the bile ducts results in severe cholestasis, which reduces the enterohepatic circulation of cobalamin. Liver dysfunction also can involve a reduction in cobalamin reserves, as the liver is the main storage site. Finally, in the course of hepatic lipidosis, liver dysfunction can lead to reduced synthesis of all transport proteins, including transcobalamin II, although it has been observed in vitro that transcobalamin II is not produced exclusively by the liver but also by other cell lines [79].

#### 4.1.8. Hyperthyroidism

A correlation between hyperthyroidism and hypocobalaminemia has been described [58], as this endocrine dysfunction directly or indirectly affects cobalamin intake, excretion, and utilization in cats. Comparing a group of hyperthyroid cats (T4 ≥ 100 nmol/L, age range 6–22 years, mean age 13 years) with a group of old euthyroid cats (T4 ≤ 40 nmol/L, age range 12–20 years, mean age 14 years), it was observed that 40% of the serum samples from the hyperthyroid group and only 25% of samples from the euthyroid group indicated hypocobalaminemia. Hyperthyroid cats seem to have increased renal loss of cobalamin and compromised intestinal absorption due to increased intestinal motility. A recent study performed in a population of cats clinically diagnosed as hyperthyroid, without other diseases, did not report the presence of hypocobalaminemia [80].

#### 4.1.9. Cardiomyopathy and Arterial Thromboembolism

McMichael et al. (2000) observed a decrease in the serum cobalamin concentration (in the normal range) in cats with cardiomyopathy and thromboembolism compared to healthy cats. That study evaluated three groups of cats: a healthy group, with mean cobalamin of 1659 ± 700 pg/mL; a cardiomyopathy group, with a mean value of 939 ± 389 pg/mL; and a cardiomyopathy–thromboembolism group, with a value of 866 ± 367 pg/mL. Low serum cobalamin concentration was also associated with left atrium enlargement. Although reduced cobalamin has been recorded in cats with cardiovascular disease, it is unclear whether it is a predisposing and causative factor or a consequence of the heart disease [81].

#### 4.1.10. Neurological Diseases

Several neurological manifestations in humans, such as dementia, are associated with cobalamin deficiency [82], while few neurological signs in cats are reported. Hypocobalaminemia status was reported in a cat affected by degenerative myelopathy [83] and in a cat with encephalopathy [84]. In the latter case report, the authors described neurological signs (dull mental status, low menace response, reduced facial sensibility, reduced postural reactions), normal urinary MMA, and hyperintense, bilaterally symmetrical, diffuse lesions on a magnetic resonance imaging (MRI) exam. After cobalamin supplementation, the cat showed improved clinical signs and MRI findings [84]. In this case report, the neurological symptoms seemed to be due to hyperammonemia rather than elevated MMA concentration.

## 5. Cobalamin Supplementation

Therapy for hypocobalaminemia mainly depends on the underlying disease [15]. Cyanocobalamin is a commonly used synthetic cobalamin compound, and naturally occurring forms of cobalamin are methylcobalamin, adenosylcobalamin, and hydroxocobalamin [85]. The use of hydroxocobalamin in cats has been described [86], and it appears to be effective in restoring cobalamin to normal ranges. In humans, hydroxocobalamin has been shown to have higher absorption and tissue retention rates than cyanocobalamin [86] but no data in cats are available.

Cobalamin supplementation (CS) can be administered either parenterally or orally (Table 1), according to the underlying disease, the level of domestication of the cat, the owner’s compliance, and the cost of therapy. Xenoulis et al. (2016) reported that in cats with exocrine pancreatic insufficiency, when cobalamin was supplemented in combination with enzyme treatment, the probability of positive response to treatment was three-fold higher [74]. Especially in the case of EPI and chronic enteropathy, cobalamin should always be measured and, if necessary, supplemented, since it is essential for the regeneration and functionality of intestinal epithelial cells. Indeed, cats with hypocobalaminemia due to gastrointestinal disease showed significant weight gain and clinical symptom improvement with CS [3,55]. Moreover, hypocobalaminemia significantly worsens the prognosis of the underlying disease, while CS can improve it [87]. Cobalamin can also be used as an appetite stimulant; anorexic cats with cobalamin deficiency often start to eat again when they are being supplemented with vitamin B12, and their appetite wanes when cobalamin is no longer administered weekly, despite having achieved a normal serum cobalamin concentration [73]. During CS, serum cobalamin concentration should always be monitored to control the achievement of the therapeutic goal. Together with serum cobalamin concentration, it can also be useful to monitor serum MMA levels, since MMA reflects the actual bioavailability of the vitamin. As cobalamin is a water-soluble vitamin, higher than normal serum cobalamin concentrations are not considered dangerous. Therefore, before supplementation is stopped, it is possible, and even recommended, to exceed normal serum cobalamin concentrations in treated patients [46]. A recognized guideline for CS does not yet exist for dogs and cats, so the recommendations are based on empirical data [15].

### 5.1. Parenteral Cobalamin Supplementation

Treatment with parenteral cobalamin is always recommended for cats with gastrointestinal disease and low serum cobalamin concentrations [37]. In some cases, 250 μg/cat, either hydroxocobalamin or adenosylcobalamin [15], is administered either subcutaneously or intramuscularly [15,37,55]. Cobalamin is administered every 7 days for 6 weeks, then every 14 days for another 6 weeks, and once a month thereafter [15,37,55]. Often the required dose depends on the primary cause of hypocobalaminemia, so serum cobalamin levels should be monitored before and after the described protocol. After administration at 14-day intervals, cobalamin should be measured 4 weeks after the last injection; if the value is within the normal range, a monthly injection follows, and if the value exceeds the reference range, the dose should be reduced and CS should be given until the primary disease is resolved [15,46]. According to Kempf et al. (2017), this CS protocol would not restore cobalamin to a normal reference range in all cats and biochemical improvements would be transient [3]. Kook et al. (2020) describe another CS protocol, in which cats with cobalamin deficiency and signs of gastrointestinal disease were administered three intramuscular injections of 300 μg hydroxocobalamin every 2 weeks. After the third injection, an initial increase in serum cobalamin concentration was observed, although the achieved level was not stable and a fourth injection was required. The latter commonly produces a decrease in serum MMA levels after 4 weeks [86].

### 5.2. Oral Cobalamin Supplementation

In recent years, it has been demonstrated that oral CS can be used successfully in dogs and cats as a less invasive alternative to parental administration [88]. Daily administration of 250 μg cyanocobalamin tablets caused a significant increase in the serum cobalamin concentration in cats with gastrointestinal disease [88]. According to Stainer (2012), oral cobalamin supplementation in cats with EPI is not effective; therefore, parenteral supplementation is necessary [73]. Oral vitamin B12 supplements complexed with IF, mainly of swine origin, are marketed for both human and veterinary use. However, it is important to point out that IF is species-specific and these vitamin B12+IF supplements have not been tested in clinical studies to support their use.

## 6. Conclusions

The suggested nutritional requirements for cobalamin have been developed from studies conducted on growing kittens. Therefore, further studies are needed to confirm that these nutritional requirements are also adequate for adult cats and pregnant and lactating queens. Moreover, determining MMA and assessing serum cobalamin can help to better identify nutritional needs by adapting them to the intracellular availability of the nutrient. Since serum MMA evaluation can better define the cobalamin status, it should be routinely performed in the clinical setting, although wide dissemination of this approach will not be possible without validating the methodologies already present in veterinary laboratories.

Due to the importance of vitamin B12 in several metabolic reactions and its role in the therapy of several diseases, vitamin status and proper supplementation should always be considered in sick cats.

Recent developments in our understanding of the gut microbiota open up new therapeutic horizons. For this reason, it would be interesting to explore the relationships between modifications in gut bacterial flora and cobalamin bioavailability and the development of chronic diseases in cats. Such studies could further speed up the collection of knowledge regarding the role and function of cobalamin.

## Figures and Tables

**Table 1:** Proposed cobalamin administration protocols.

Parenteral Administration ^a^ [15,37,55]	Parenteral Administration ^a^ [86]	Oral Administration ^a^ [88]
250 μg SC/IM every 7 days for 6 weeks, then 250 μg SC/IM every 14 days for 6 weeks	300 μg hydroxocobalamin IM every 14 days 3–4 times	250 μg cyanocobalamin every day until serum concentration exceeds range
If value is in normal range, 250 μg SC/IM once a month; if value exceeds range, reduce dose and continue treatment until primary disease is resolved		

μg, micrograms; SC, subcutaneous; IM, intramuscular. ^a^ Serum cobalamin status should be checked during supplementation; checking at least monthly should be recommended.

## Data Availability

No new data were created or analyzed in this study. Data sharing is not applicable to this article.
